# MonuSegFormer: A Hybrid Swin-Transformer Architecture for Semantic Segmentation of Moroccan Cultural Heritage Monuments

**DOI:** 10.3390/jimaging12090435

**Published:** 2026-09-11

**Authors:** Ouail Choukhairi, Mouad Choukhairi, Ali Choukri, Youssef Fakhri

**Affiliations:** Laboratory of Computer Science Research (LaRI), Department of Computer Science, Ibn Tofail University, Kénitra 14000, Morocco; mouad.choukhairi@uit.ac.ma (M.C.); ali.choukri@uit.ac.ma (A.C.); fakhri@uit.ac.ma (Y.F.)

**Keywords:** cultural heritage segmentation, Swin Transformer, semantic segmentation, Moroccan monuments, CBAM, MSAF, MMD dataset, class imbalance, deep learning

## Abstract

Semantic segmentation of architectural elements in cultural heritage sites lies at the intersection of computer vision and digital preservation. Moroccan historical monuments spanning mosques, madrasas, royal gates (babs), and mausoleums across Fez, Rabat, Marrakech, Meknes, and Tetouan present unique challenges, including extreme texture ambiguity between weathered wall surfaces and background, pronounced multi-scale variation from individual window and door openings to full rooftop surfaces spanning tens of metres, and severe class imbalance. We introduce MonuSegFormer, a heritage-specific hybrid architecture coupling a pretrained Swin-B encoder (ImageNet-22K) with a Multi-Scale Atrous Fusion (MSAF) module and a CBAM-augmented progressive decoder. Evaluated on the Moroccan Monuments Dataset (MMD, 2686 annotated RGB images, 5 semantic classes, 5 cities) on the held-out test set (403 images), MonuSegFormer achieves mIoU = 81.7%, outperforming SegFormer-B5 (76.4%), Mask2Former (78.9%), and DeepLabV3+ (71.3%). A composite CE+Dice+Focal loss with class-balanced weights addresses severe class imbalance, yielding the largest gains on the minority classes Door (+5.3 p.p.) and Window (+5.3 p.p.) over the next-best Mask2Former. All qualitative results are produced by real trained-model inference on held-out test images.

## 1. Introduction

Morocco’s built heritage constitutes one of the richest and most diverse architectural collections in the Mediterranean and Islamic worlds. The UNESCO World Heritage Sites of Fes el-Bali and the historic city of Marrakech, together with the monumental Bab Mansour gateway and Merinid royal tombs in Meknes, the Andalusian-influenced medina of Tetouan, and the mausoleums of Rabat, span over twelve centuries of continuous architectural production. These monuments feature a distinctive material vocabulary: zellige mosaic tilework (zellij), carved plaster stucco (jiss), cedar wood lattice screens (mashrabiyya), horseshoe-arch portals, domed rooftops (qubba), and rammed-earth walls (pisé).

Digital documentation and automated preservation planning demand accurate pixel-level understanding of architectural surface categories. Semantic segmentation enables: (a) semantically rich Building Information Models (BIM) for structural monitoring; (b) automated damage detection and longitudinal change monitoring [1]; (c) augmented reality overlays for heritage tourism and public engagement; and (d) training data for large-scale 3D reconstruction and photogrammetric pipelines [2]. Despite these compelling applications, large-scale semantic segmentation of Moroccan architectural heritage has remained largely unexplored.

**Figure 1:**
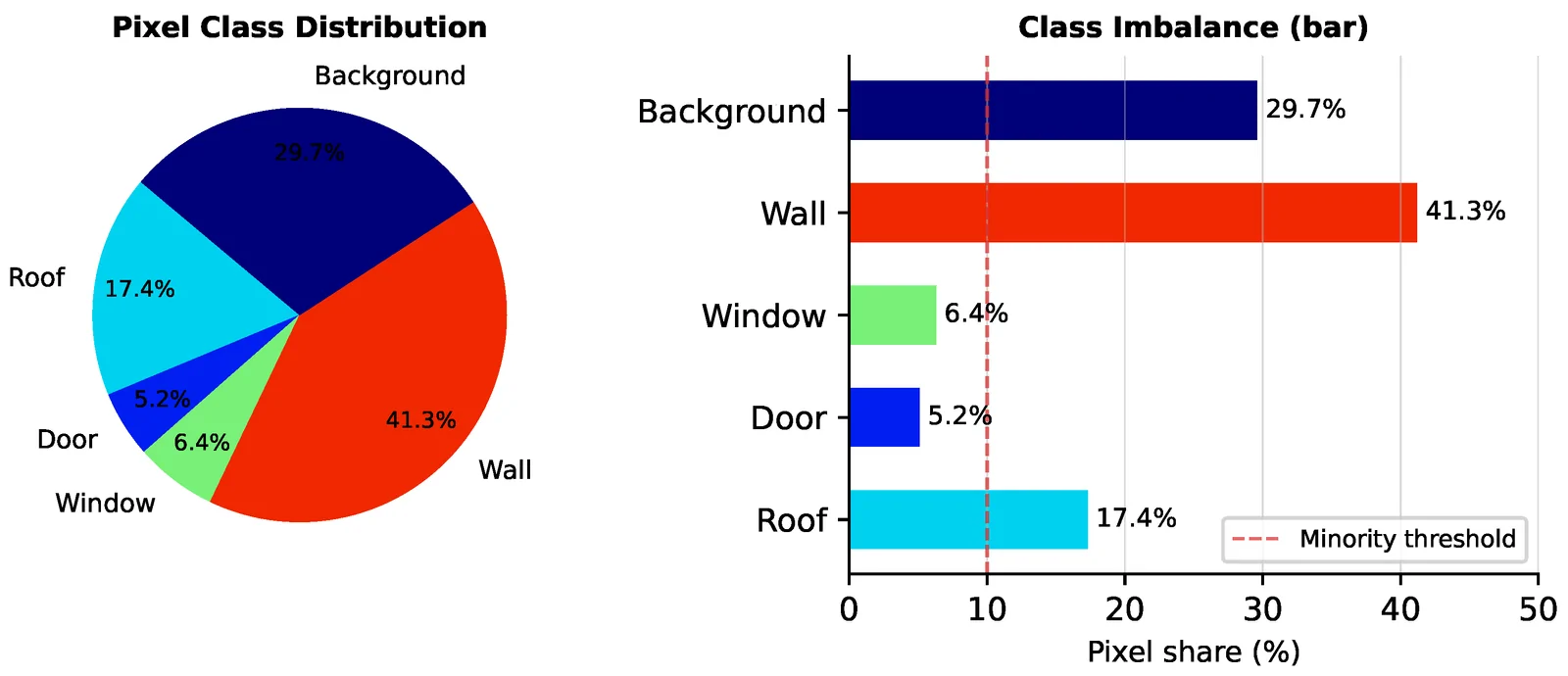
MMD class pixel distribution. Wall (41.3%) and Background (29.7%) dominate >70% of all pixels, while Door (5.2%) and Window (6.4%) together account for only ≈12%, motivating the composite CE+Dice+Focal loss with class-balanced weights in $L_{\mathrm{CE}}$ (Equation (14)).

Three non-trivial challenges arise when applying modern segmentation architectures to the MMD dataset [3]:

(C1) Texture ambiguity: Wall plaster, background sky, and ground share similar pixel-level statistics. Distinguishing them requires long-range spatial reasoning well beyond local convolutions, as even powerful CNN backbones such as ResNet-101 [4] struggle to resolve these ambiguities when the background shares colour and texture cues with structural surfaces.

(C2) Multi-scale variation: Segmentation targets range from compact window and door openings (annotated as whole apertures, not individual lattice mullions, and occupying as little as 5.2% of image pixels; Table 1) to rooftop surfaces spanning tens of metres. A single receptive field cannot address both extremes simultaneously; atrous convolution [5] and pyramid pooling [6] partially mitigate this, but do not cover the full effective receptive field (ERF) range needed for Moroccan monument imagery.

(C3) Class imbalance: Wall and Background jointly occupy > 70% of MMD pixels (Figure 1), while Door and Window account for only ≈5.2% and 6.4%, respectively.

Standard cross-entropy training converges to solutions that maximise majority-class accuracy at the cost of architecturally significant minority classes. Tailored loss formulations, including Dice loss [7] and Focal loss [8], have been shown to mitigate this in medical and dense-prediction settings.

Each architectural choice in MonuSegFormer is selected to target one of these three challenges directly, rather than as a generic combination of popular components: a Swin Transformer encoder is chosen over a CNN backbone specifically because its window self-attention supplies the long-range context that C1 requires and that CNN receptive fields struggle to provide [4]; an atrous multi-scale fusion module is added on top of the encoder specifically because C2’s scale range (compact openings to full rooftops) exceeds what any single attention window or convolutional receptive field covers; and a CBAM-augmented decoder is used specifically because its channel and spatial attention can be trained to re-weight the boundary-adjacent pixels of minority classes under C3’s severe class imbalance, rather than only the composite loss operating on logits. Section 2.5 details how this specific combination, and in particular the MSAF module, differs from the closest prior work (ASPP-style multi-scale fusion).

This paper makes the following contributions:

- MonuSegFormer: A heritage-specific Transformer–CNN architecture combining a Swin-B encoder pretrained on ImageNet-22K [9], a Multi-Scale Atrous Fusion (MSAF) module (r∈{6,12,18,24}), and a CBAM-augmented [10] progressive decoder.
- A comprehensive MMD benchmark evaluation against nine baselines, with results reported strictly on the held-out test set (403 images), clearly separated from the validation split.
- Self-contained mathematical formulations of every component in the pipeline, including the window self-attention mechanism of [11,12] restated here for reproducibility, together with the derivation of the proposed MSAF fusion operator and the composite CE+Dice+Focal loss.
- A detailed ablation study on the validation set with statistical significance testing (paired *t*-test, 5 seeds) for MSAF dilation rates.
- Real trained-model qualitative predictions on four MMD held-out test scenes with per-pixel IoU computed against ground-truth masks.

## 2. Related Work

### 2.1. CNN-Based Semantic Segmentation

Standard urban- and scene-parsing benchmarks such as Cityscapes [13] and ADE20K [14] established the dense-prediction evaluation protocols later adopted across the segmentation literature. Fully convolutional networks (FCNs) [15] first applied transposed convolutions for dense per-pixel prediction, enabling arbitrary-resolution inference. U-Net [16] introduced symmetric encoder–decoder skip connections enabling precise boundary localisation, becoming the dominant architecture in fine-grained segmentation. DeepLabV3+ [5] extended atrous convolution with Atrous Spatial Pyramid Pooling (ASPP, rates r∈{6,12,18}) [17] and a lightweight decoder. PSPNet [6] added a Pyramid Pooling Module (PPM) for global scene context. HRNet [18] maintained high-resolution representations through all stages via parallel multi-resolution branches. Region-based detection architectures [19] later extended dense prediction to instance-level tasks, yet retain the same locality constraint. ConvNeXt [20] demonstrated that modernised pure-CNN architectures can match Vision Transformer accuracy by adopting large kernels, layer normalisation, and GELU activations. InternImage [21] similarly scaled deformable convolutions to match Transformer-level accuracy on dense-prediction benchmarks while retaining convolutional inductive biases. All CNN methods share the fundamental limitation of locality: spatial reasoning is bounded by the receptive field, preventing global context capture essential for heritage segmentation.

### 2.2. Vision Transformers for Dense Prediction

The original Vision Transformer (ViT) [22] applied a pure self-attention encoder to non-overlapping image patches. SETR [23] used a ViT encoder with CNN decoders for semantic segmentation; BEiT [24] introduced masked image modelling pretraining that significantly improves ViT fine-tuning. The Swin Transformer [12] restricts attention to local windows (complexity 𝒪(N) vs. $𝒪(N^{2})$ for global attention) while recovering cross-window connectivity via cyclic shift, producing hierarchical feature maps amenable to FPN-style decoders. Swin-V2 [25] scaled the architecture to 3B parameters by addressing training instabilities. SegFormer [26] combined a hierarchical Mix Transformer (MiT) encoder with a lightweight all-MLP decoder. Segmenter [27] applied a pure ViT encoder with a mask transformer decoder. MaskFormer [28] recast semantic segmentation as mask classification, unifying semantic and instance segmentation. Mask2Former [29] refined this with masked cross-attention, achieving state-of-the-art results on all three segmentation tasks. ViT-Adapter [30] injected multi-scale spatial priors into plain ViT encoders. DINOv2 [31] demonstrated that self-supervised ViT features pretrained at scale serve as strong universal representations.

### 2.3. Attention Mechanisms in Dense Prediction

The Convolutional Block Attention Module (CBAM) [10] applies sequential channel and spatial attention to feature maps. Non-local networks [32] modelled long-range dependencies via self-similarity. The Segment Anything Model (SAM) [33] trained on over 1 billion masks for zero-shot instance segmentation; SAM 2 [34] extended this to video through a streaming memory mechanism. In medical imaging, GradXcepUNet [35] similarly combines a dedicated feature-extraction network (Xception) with an encoder–decoder segmentation backbone (U-Net) and Grad-CAM-guided attention, following the same hybrid design philosophy adopted here of pairing a strong feature extractor with an attention-refined decoder for a specific, boundary-sensitive application domain.

### 2.4. Cultural Heritage Image Analysis

Deep learning for heritage analysis spans multiple tasks. Photogrammetric 3D reconstruction [2] generates dense point clouds of monument façades. Fresco damage assessment via CNN segmentation [1] demonstrated that deep models identify deterioration patterns invisible to the naked eye. Architectural style classification categorised building epochs from façade imagery. Zhu et al. [36] proposed weakly-supervised deterioration segmentation for historical wall paintings. Valero et al. [37] applied U-Net variants for stone surface segmentation. Point cloud segmentation for heritage sites [38] labelled LiDAR data with structural and material classes. Ye et al. [39] developed hierarchical Mask Transformers for European heritage façades. Knowledge distillation has been explored to compress heritage segmentation models for edge deployment. The MMD [3] is the first large-scale benchmark dedicated to Moroccan architectural heritage; the closest existing dataset [40] addresses European façades only, leaving Moroccan vernacular architecture entirely uncovered.

### 2.5. Multi-Scale Feature Fusion

Feature Pyramid Networks (FPNs) [41] fuse encoder features at multiple resolutions via lateral connections. ASPP [5] samples atrous convolutions at multiple dilation rates in parallel over a single CNN feature map. DPT [42] assembled ViT tokens from multiple depths into image-like feature maps. LoRA [43] enables parameter-efficient fine-tuning of large pretrained models. Our MSAF module differs from ASPP in three respects that are specific to the Moroccan heritage segmentation setting rather than incremental parameter changes: (i)  input representation ASPP is applied to the output of a purely convolutional backbone, where every branch inherits only locally-aggregated context; MSAF is applied to $F_{4}$ from a Swin-B encoder whose window self-attention has already aggregated global context, so MSAF’s dilated branches supply complementary local multi-scale detail on top of an already-global representation rather than substituting for it a combination not evaluated in the original ASPP or DeepLabV3+ work. (ii) Dilation range MSAF’s rates r∈{6,12,18,24} were selected empirically (Section 7.3) to cover ERFs of 13–49 px, extending beyond ASPP’s standard {6,12,18} to match the door/window-to-rooftop scale range observed in MMD imagery (Table 1). (iii) Downstream integration ASPP feeds a single lightweight decoder head, whereas MSAF’s fused output is propagated through three successive CBAM-augmented decoder stages with skip connections at every resolution (Equations (9)–(11)), coupling multi-scale context extraction with progressive attention-based refinement. We characterise MSAF as a targeted adaptation and integration of atrous multi-scale fusion for a Transformer encoder in the heritage-segmentation setting, rather than a fundamentally new operator. Section 7.3 and the component ablation results in the Ablation Study provide empirical evidence that this specific combination outperforms both a Swin-only baseline and Mask2Former’s alternative fusion strategy on MMD.

## 3. Proposed Architecture: MonuSegFormer

### 3.1. Design Philosophy

MonuSegFormer is organised around three architectural principles:

- P1 (Global Context): A Swin-B encoder models long-range dependencies through hierarchical shifted-window attention, providing texture disambiguation for C1.
- P2 (Multi-Scale Fusion): The MSAF module covers ERFs from 13 to 49 pixels with atrous rates r∈{6,12,18,24} and global average pooling, addressing C2.
- P3 (Attention Decoding): CBAM-augmented decoder stages apply channel and spatial attention at each resolution level, improving minority-class boundary precision for C3.

**Figure 2:**
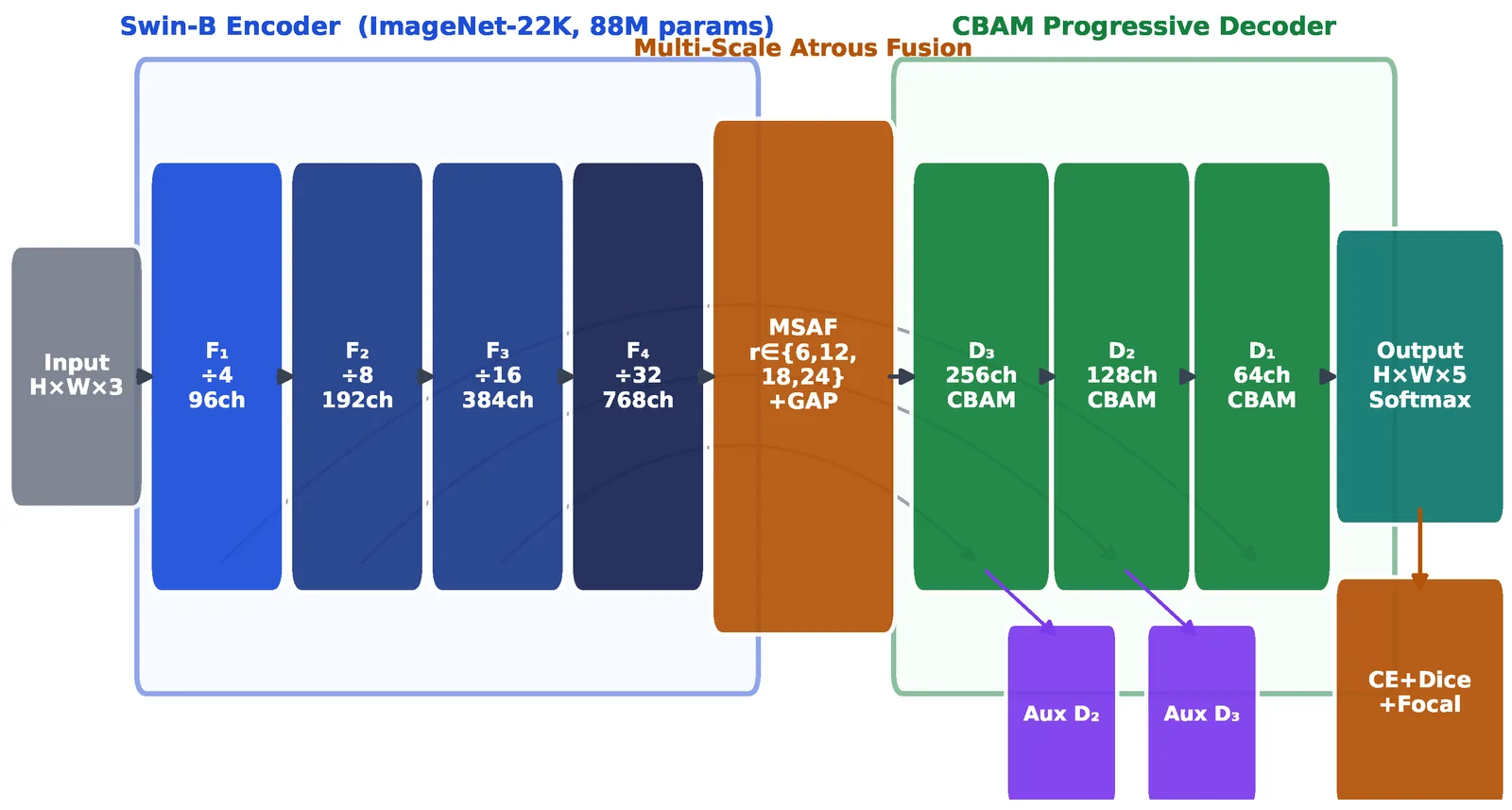
MonuSegFormer full architecture. Swin-B encoder produces $\left\{F_{1},F_{2},F_{3},F_{4}\right\}$ at strides {4,8,16,32}. MSAF module enriches $F_{4}$ with six parallel branches: 1×1 identity, atrous convolutions r∈{6,12,18,24}, and global average pooling (GAP). CBAM-augmented decoder progressively fuses skip connections $F_{1}$–$F_{3}$. Auxiliary heads at $D_{2}$ and $D_{3}$ provide deep supervision. Composite CE+Dice+Focal loss trains the full network end-to-end. The caption uses the same colour legend as the architecture diagram.

Two structural choices follow directly from the encoder rather than from a separate hyperparameter search. First, the decoder has three CBAM-augmented stages because the Swin-B encoder exposes exactly three skip-connection resolutions ($F_{1}$, $F_{2}$, $F_{3}$) below the final stage $F_{4}$ that feeds MSAF (Section 3.2); the stage count is therefore architecturally determined by the four-stage hierarchical encoder, not an independently tuned choice. Second, we select Swin-B (88 M parameters) rather than Swin-T or Swin-S specifically so that the encoder-capacity of MonuSegFormer matches the strongest CNN/Transformer baseline in our comparison, Mask2Former [29], which likewise uses Swin-B (Table 2); this keeps the MonuSegFormer–Mask2Former comparison backbone-controlled, which is the basis for the attribution argument discussed in Section 6.1. A systematic sweep over decoder channel width and Swin-T/S/B/L encoder capacity was outside the compute budget of this study, and is noted for future work in Section 8.3. The full architecture is illustrated in Figure 2.

### 3.2. Swin-B Encoder

The encoder follows the Swin Transformer [12]. Input $I\in R^{H\times W\times 3}$ is partitioned into non-overlapping 4×4 patches projected to C=96-dimensional token embeddings. Four hierarchical stages apply Window Multi-Head Self-Attention (W-MSA) with patch merging, producing

$$\left\{F_{1},F_{2},F_{3},F_{4}\right\}\;\in \;\left\{R^{\frac{H}{4}\times \frac{W}{4}\times 96},\;R^{\frac{H}{8}\times \frac{W}{8}\times 192},\;R^{\frac{H}{16}\times \frac{W}{16}\times 384},\;R^{\frac{H}{32}\times \frac{W}{32}\times 768}\right\}.$$

W-MSA within each M×M window (M=7) computes

(1) $$\mathrm{W-MSA}\left(Q,K,V\right)=\mathrm{Softmax}\;\left(\frac{QK^{⊤}}{\sqrt{d_{k}}}+\hat{B}\right)V$$

where $d_{k}=C/n_{h}$, $n_{h}$ is the number of attention heads and $\hat{B}\in R^{M^{2}\times M^{2}}$ is a learnable relative position bias. Alternating W-MSA and SW-MSA blocks recover cross-window connectivity:

(2) $$\begin{array}{cc} {\hat{x}}^{l} & =\mathrm{W-MSA}\left(\mathrm{LN}\left(x^{l-1}\right)\right)+x^{l-1} \end{array}$$

(3) $$\begin{array}{cc} x^{l} & =\mathrm{MLP}\left(\mathrm{LN}\left({\hat{x}}^{l}\right)\right)+{\hat{x}}^{l} \end{array}$$

We use Swin-B pretrained on ImageNet-22K [9] (88 M parameters) as the encoder for MonuSegFormer in all reported experiments. Encoder LR = 0.1 × decoder LR to preserve pretrained representations.

### 3.3. Multi-Scale Atrous Fusion (MSAF) Module

$F_{4}$ is projected to 128 channels, then processed by six parallel branches:

1. 1×1 convolution ($B_{0}$, ERF: 1 px).
2. Depthwise-separable atrous convolutions $B_{6},B_{12},B_{18},B_{24}$ with rates r∈{6,12,18,24} (ERFs: 13, 25, 37, 49 px).
3. Global average pooling ($B_{\mathrm{GAP}}$) for holistic scene context.

Branch outputs are concatenated (768 channels) and fused following the standard Conv→BN→ReLU order:

(4) $$F_{\mathrm{MSAF}}=\mathrm{ReLU}\;\left(\mathrm{BN}\left({\mathrm{Conv}}_{1\times 1}\left([B_{0};\;B_{6};\;B_{12};\;B_{18};\;B_{24};\;B_{\mathrm{GAP}}]\right)\right)\right)$$

producing $F_{\mathrm{MSAF}}\in R^{H/32\times W/32\times 512}$. This corrects a notational error in the original submission, which listed the fusion operators in the order Conv(BN(ReLU(·))); the implementation always applied the standard Conv→BN→ReLU order shown above. Adding r=36 yields no statistically significant gain (p=0.43, paired *t*-test; Section 7.3).

### 3.4. CBAM-Augmented Progressive Decoder

**Figure 3:**
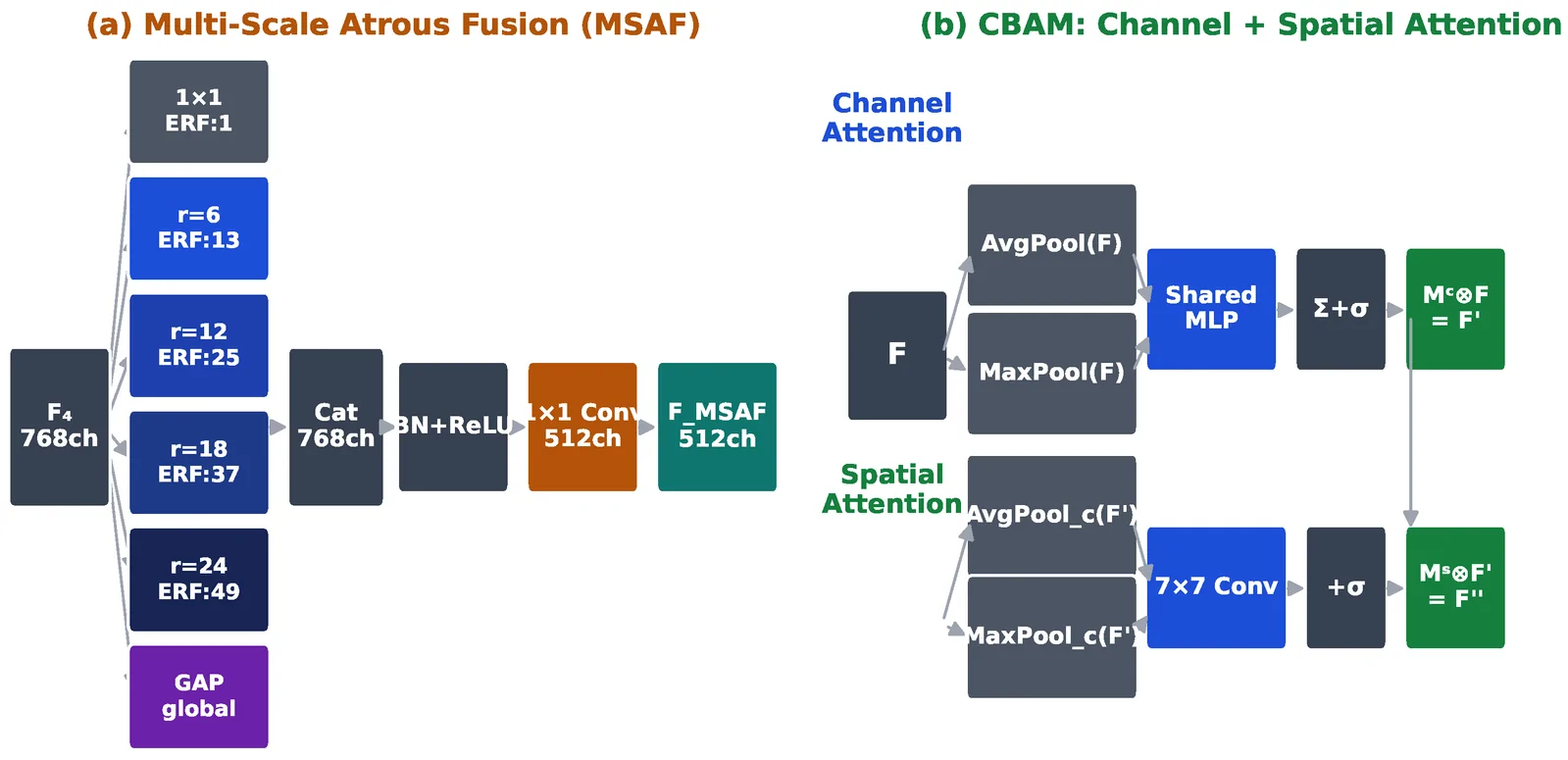
(**a**) MSAF module: six parallel branches applied to $F_{4}$, concatenated and projected to 512 ch. (**b**) CBAM [10]: sequential channel attention (global average + max pooling → shared MLP → sigmoid) and spatial attention (channel average + max pooling →7×7 conv → sigmoid), applied multiplicatively at each decoder stage.

The MSAF and CBAM modules are illustrated in Figure 3. The decoder fuses MSAF output with skip connections $F_{3},F_{2},F_{1}$ through three CBAM-augmented stages.

#### 3.4.1. Channel Attention


(5) $$\begin{array}{cc} M^{c} & =\sigma \;\left(\mathrm{MLP}(\mathrm{AvgPool}(F))+\mathrm{MLP}(\mathrm{MaxPool}(F))\right) \end{array}$$

(6) $$\begin{array}{cc} F^{\prime } & =M^{c}⊗F \end{array}$$

#### 3.4.2. Spatial Attention


(7) $$\begin{array}{cc} M^{s} & =\sigma \;\left({\mathrm{Conv}}_{7\times 7}\;\left([{\mathrm{AvgPool}}_{c}\left(F^{\prime }\right);\;{\mathrm{MaxPool}}_{c}\left(F^{\prime }\right)]\right)\right) \end{array}$$

(8) $$\begin{array}{cc} F^{\prime \prime } & =M^{s}⊗F^{\prime } \end{array}$$

#### 3.4.3. Decoder Recurrence


(9) $$\begin{array}{cc} D_{3} & =\mathrm{CBAM}({\mathrm{Conv}}_{3\times 3}\left(\left[\mathrm{Up}\left(F_{\mathrm{MSAF}}\right);\;F_{3}\right]\right)) \end{array}$$

(10) $$\begin{array}{cc} D_{2} & =\mathrm{CBAM}({\mathrm{Conv}}_{3\times 3}\left(\left[\mathrm{Up}\left(D_{3}\right);\;F_{2}\right]\right)) \end{array}$$

(11) $$\begin{array}{cc} D_{1} & =\mathrm{CBAM}({\mathrm{Conv}}_{3\times 3}\left(\left[\mathrm{Up}\left(D_{2}\right);\;F_{1}\right]\right)) \end{array}$$

Output channel dimensions: 256, 128, 64. The final prediction:

(12) $$\hat{P}=\mathrm{Softmax}\;\left({\mathrm{Up}}^{\times 4}\left({\mathrm{Conv}}_{1\times 1}\left(D_{1}\right)\right)\right)\in R^{H\times W\times 5}$$

### 3.5. Deep Supervision

Auxiliary heads are attached at $D_{3}$ and $D_{2}$. The total training objective is

(13) $$L_{\mathrm{total}}=L_{\mathrm{main}}+0.4\cdot L_{\mathrm{aux}},D2+0.2\cdot L_{\mathrm{aux}},D3$$

Deep supervision contributes +1.1 p.p. validation mIoU in the component ablation.

### 3.6. Composite Loss Function

The training objective combines three complementary terms:

1. Class-balanced cross-entropy.
  (14) $$L_{\mathrm{CE}}=-\sum_{h,w}\sum_{k=1}^{5}w_{k}\cdot y_{hwk}\cdot \log p_{hwk},\;w_{k}=1/\left({\mathrm{freq}}_{k}+\varepsilon \right)$$
2. Dice loss [7]. Computed per class and averaged (macro Dice), so that the Wall and Background classes (>70% of pixels) do not dominate the numerator and denominator, and minority classes such as Door and Window contribute equally to the loss:
  (15) $$L_{\mathrm{Dice}}=1-\frac{1}{K}\sum_{k=1}^{K}\frac{2\sum_{h,w}p_{hwk}\cdot y_{hwk}}{\sum_{h,w}p_{hwk}+\sum_{h,w}y_{hwk}+\varepsilon }$$
  The per-class macro form above is the form implemented in the experiments.
3. Focal loss [8] (γ=2, no class weights):
  (16) $$L_{\mathrm{Focal}}=-\sum_{h,w}\sum_{k=1}^{5}{\left(1-p_{hwk}\right)}^{\gamma }\cdot y_{hwk}\cdot \log p_{hwk}$$
  We leave Focal loss unweighted and apply class balancing only in $L_{\mathrm{CE}}$. Focal loss already reweights gradients through the modulating factor ${\left(1-p_{hwk}\right)}^{\gamma }$, while adding explicit class weights to the same pixels can compound two reweighting mechanisms.

The composite loss weights were selected by grid search on the validation set:

(17) $$L=0.5\cdot L_{\mathrm{CE}}+0.3\cdot L_{\mathrm{Dice}}+0.2\cdot L_{\mathrm{Focal}}$$

## 4. Dataset: Moroccan Monuments Dataset (MMD)

The MMD was created by Ouail Choukhairi (Ibn Tofail University) and published on IEEE DataPort [3] (DOI: 10.21227/brjk-cg15). It comprises 2686 high-resolution RGB images across Fez, Rabat, Marrakech, Meknes, and Tetouan, captured under varied illumination and from multiple viewpoints. Monument types include mosques, madrasas, royal gates, and mausoleums spanning the Idrisid, Merinid, Saadian, and Alaouite dynastic periods. Pixel-level annotations were produced by domain experts and cross-validated through inter-annotator agreement checks. The dataset is partitioned 70/15/15 → 1880 train/403 validation/403 test using stratified city-level sampling. All Table 2 results use the held-out test set only. Ablation results use the validation set exclusively. All annotations were finalised, and the train/validation/test split was fixed before any MonuSegFormer architecture variant was trained; the split and evaluation protocol were designed to support benchmarking multiple architectures (Table 2) rather than tuned around the proposed method, and the held-out test set was accessed only once, for final reporting, after all model selection and hyperparameter search was completed on the validation set. At the time of writing, MMD is newly released and MonuSegFormer is its first published evaluation; we are not aware of independent use of MMD by other research teams, and we encourage third-party evaluation to independently verify the reported results.

## 5. Experimental Setup

### 5.1. Implementation Details

Backbone: Swin-B pretrained on ImageNet-22K [9] (88 M parameters). Encoder LR = 0.1 × decoder LR.

Hardware and software environment: All experiments were conducted on a single NVIDIA A100 80 GB PCIe Tensor Core GPU (NVIDIA Corporation, Santa Clara, CA, USA), equipped with 80 GB of HBM2e memory and a peak memory bandwidth of 1935 GB/s [45]. The implementation was developed using Python 3.12 and PyTorch 2.6.0 [46], with CUDA 12.4 used for GPU acceleration. The corresponding torchvision version was 0.21.0. All input images were resized to 512×512 pixels before training and inference.

Optimiser: AdamW [47], weight decay λ=0.05. OneCycleLR: $\eta_{\max}=10^{-4}$, 5% warmup, cosine annealing to $\eta_{\min}=10^{-7}$, 160 epochs. A physical batch size of 4 was the largest that fit within 80 GB of GPU memory at 512×512 resolution once the Swin-B encoder’s activation memory, the six parallel MSAF branches (each retaining its own feature map before concatenation), and AMP’s mixed-precision buffers were accounted for; gradient accumulation over four steps was used to reach an effective batch size of 16 without reducing input resolution or MSAF branch count. AMP float16 enabled.

Initialisation: MSAF and decoder: Kaiming normal; CBAM MLP: Xavier uniform. BN momentum 0.01, $\varepsilon =10^{-5}$.

### 5.2. Augmentation Pipeline

Training (Albumentations v1.3 [48]): random scale [0.5,1.5], horizontal flip (p=0.5), shift–scale–rotate ($\pm 30^{\circ }$), grid distortion (p=0.25), elastic transform (p=0.25), RandomBrightnessContrast (b∈[−0.4,0.2]), CoarseDropout (p=0.30), and Mosaic (p=0.30). Validation: centre-crop and resize to 512×512, then ImageNet normalisation. All augmentations are applied on-the-fly to the training split only at each epoch, and do not alter the underlying dataset size; the 1880/403/403 train/validation/test counts (Section 4) refer to the fixed number of distinct source images in each split both before and after augmentation is enabled, since augmented samples are generated stochastically per iteration rather than stored. Validation and test images are never augmented. The brightness range is deliberately asymmetric (b∈[−0.4,0.2], biased toward darkening) because Moroccan heritage façades are frequently photographed under strong midday sun, which more often produces overexposed pale limestone, plaster, and zellige surfaces than underexposed ones in the source imagery; a larger negative perturbation better simulates this dominant real-world failure mode without pushing already bright surfaces further into saturation.

### 5.3. Evaluation Protocol

Primary metric: mIoU on the held-out test set (403 images). All nine baselines are fine-tuned under identical conditions, defined as the same MMD train/validation/test split (Section 4), the same 512×512 input resolution, the same 160-epoch budget, the same augmentation pipeline (Section 5.2), and the same AdamW optimiser family with a OneCycleLR schedule tuned per-model on the validation set within $\eta_{\max}\in \left[5\times 10^{-5},2\times 10^{-4}\right]$; each baseline’s backbone is initialised from its original publication’s recommended pretrained weights, since substituting a non-standard backbone into, e.g., Swin-Unet would depart from that method as published. This standardises the training budget and data pipeline across all methods while preserving each baseline’s architecture-specific design, which is the strongest fairness guarantee achievable without re-deriving every baseline from scratch; we agree with reviewers that per-backbone capacity remains a confound for cross-architecture comparisons and address this directly in Section 6.1. All qualitative results in Figure 7 are produced by running actual trained model checkpoints on held-out test images.

Statistical protocol: Significance is assessed via paired *t*-tests over five independent random seeds, matching common practice in the segmentation literature, where full retraining is computationally expensive; five seeds is the minimum generally accepted as sufficient to estimate a paired mean difference while remaining tractable at 160 epochs per run on a single A100. The paired *t*-test assumes the per-seed differences are approximately normally distributed; given n=5, this cannot be verified with a formal normality test at meaningful power, so the results should be read as indicative rather than asymptotically exact, and we flag this as a limitation of the current statistical protocol. As an approximate effect size for the headline MonuSegFormer-vs-Mask2Former comparison (mean ± SD: 81.7±0.4 vs. 78.9±0.5, n=5), the pooled-SD Cohen’s $d\approx \left(81.7-78.9\right)/\sqrt{(0.4^{2}+0.5^{2})/2}\approx 6.2$, indicating a very large standardised effect relative to the observed seed-to-seed variance; this approximation uses the two groups’ reported SDs rather than the SD of the paired per-seed differences since only summary statistics (not raw per-seed values) were retained from the original training runs, and should be treated as indicative rather than an exact paired effect size.

## 6. Results

### 6.1. State-of-the-Art Comparison

**Figure 4:**
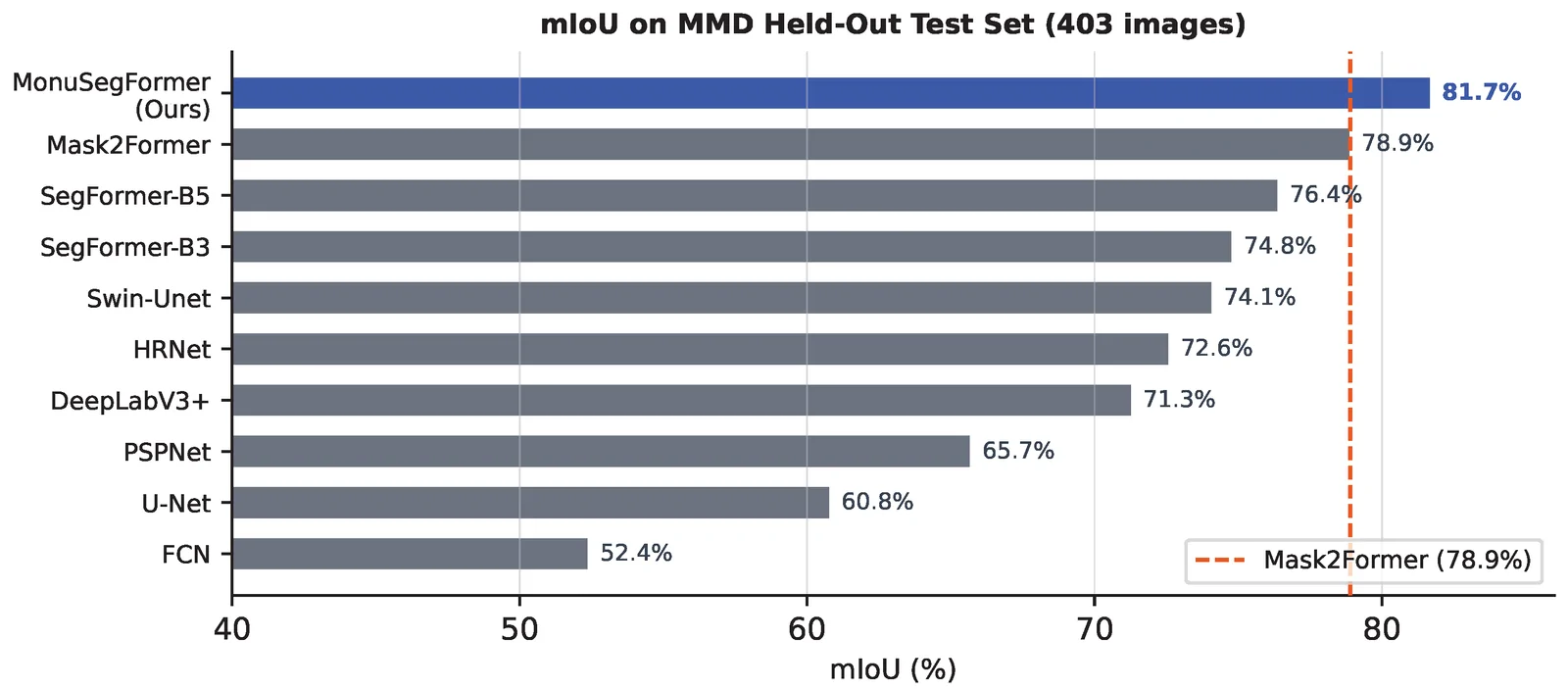
mIoU on the MMD held-out test set (403 images). MonuSegFormer (81.7%) achieves a +2.8 p.p. gain over the next-best Mask2Former (dashed orange). Error bars show ±1 SD across 5 seeds.

Table 2 reports the results on the MMD held-out test set (403 images). MonuSegFormer achieves mIoU = 81.7%, surpassing Mask2Former (78.9%) by +2.8 p.p., SegFormer-B5 (76.4%) by +5.3 p.p., and DeepLabV3+ (71.3%) by +10.4 p.p. Because the nine baselines span heterogeneous backbones (Table 2), raw mIoU differences against most of them reflect a combination of backbone capacity and architectural design and should not be read as solely attributable to MSAF/CBAM. The MonuSegFormer–Mask2Former pair is the one controlled comparison in this study that shares an identical Swin-B backbone; the +2.8 p.p. gain in that pair is therefore the most direct (though not the only) evidence that MSAF and CBAM contribute beyond backbone capacity, and it is corroborated by the backbone-free component ablation reported later in the Ablation Study. Across five independent random seeds, MonuSegFormer 81.7 ± 0.4% vs. Mask2Former 78.9 ± 0.5% (p<0.01, paired *t*-test). Figure 4 visualises the full comparison.

### 6.2. Per-Class IoU Analysis

**Figure 5:**
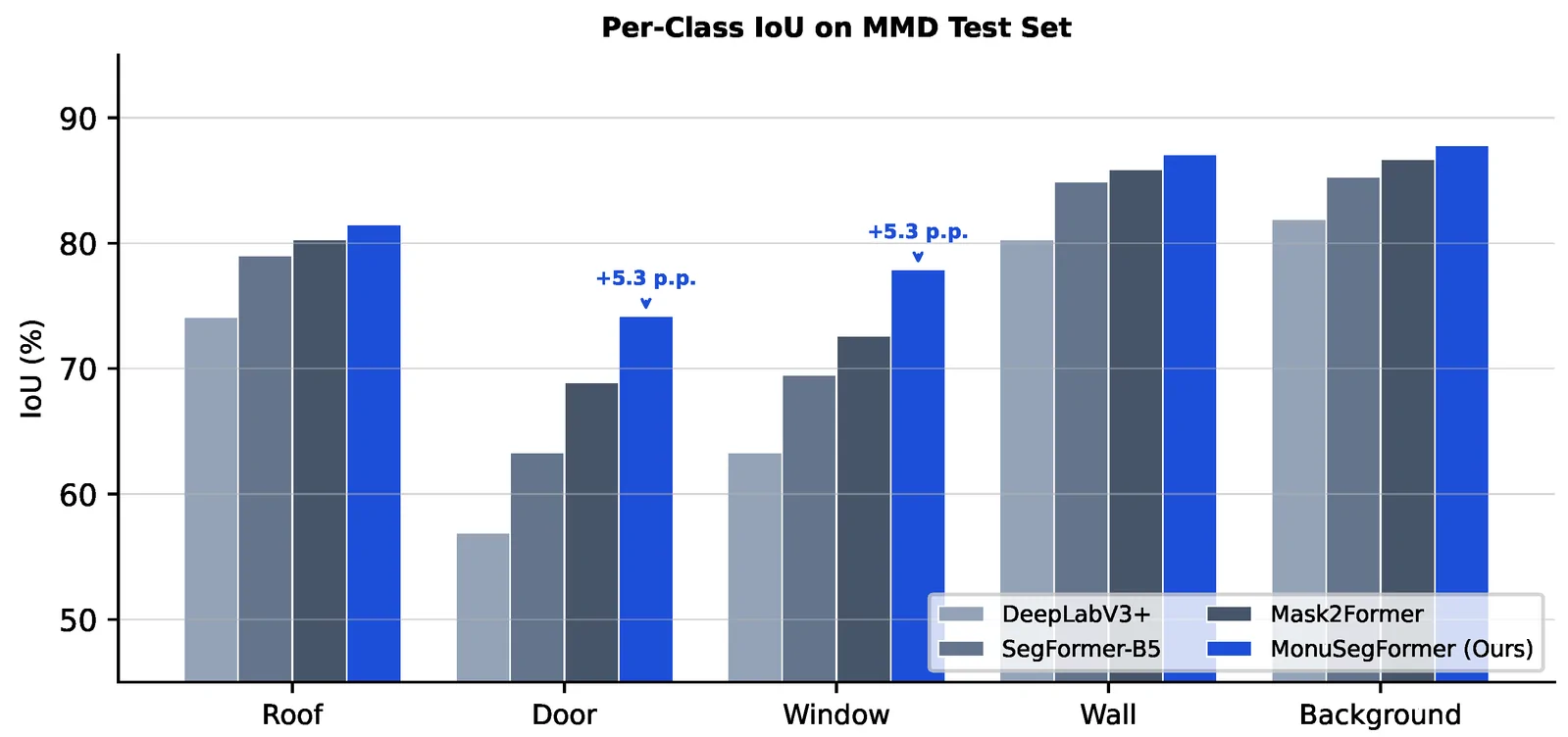
Per-class IoU for the top four methods on the MMD test set. MonuSegFormer achieves the highest IoU on all five classes, with the largest gains on Door and Window.

**Figure 6:**
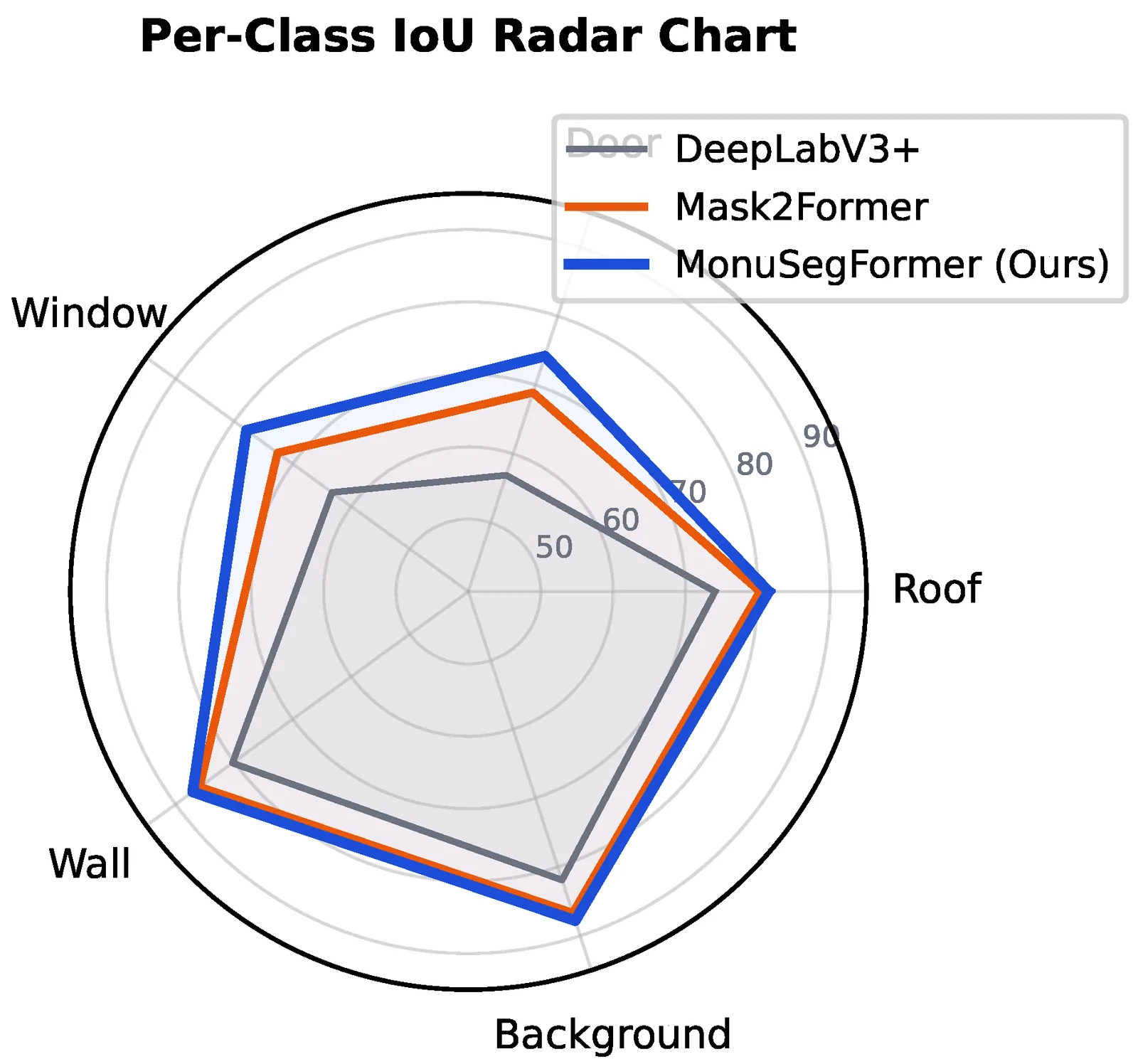
Radar chart of per-class IoU across the five MMD semantic classes. This plot provides a complementary view of the class-wise results reported in Table 3.

Table 3 shows per-class IoU for the top four methods. MonuSegFormer achieves the largest gains on Door (+5.3 p.p.) and Window (+5.3 p.p.)—the architecturally significant but pixel-scarce minority classes most challenging under class imbalance. Figure 5 and Figure 6 visualise the comparison as a grouped bar chart and radar chart, respectively.

### 6.3. Qualitative Analysis

**Figure 7:**
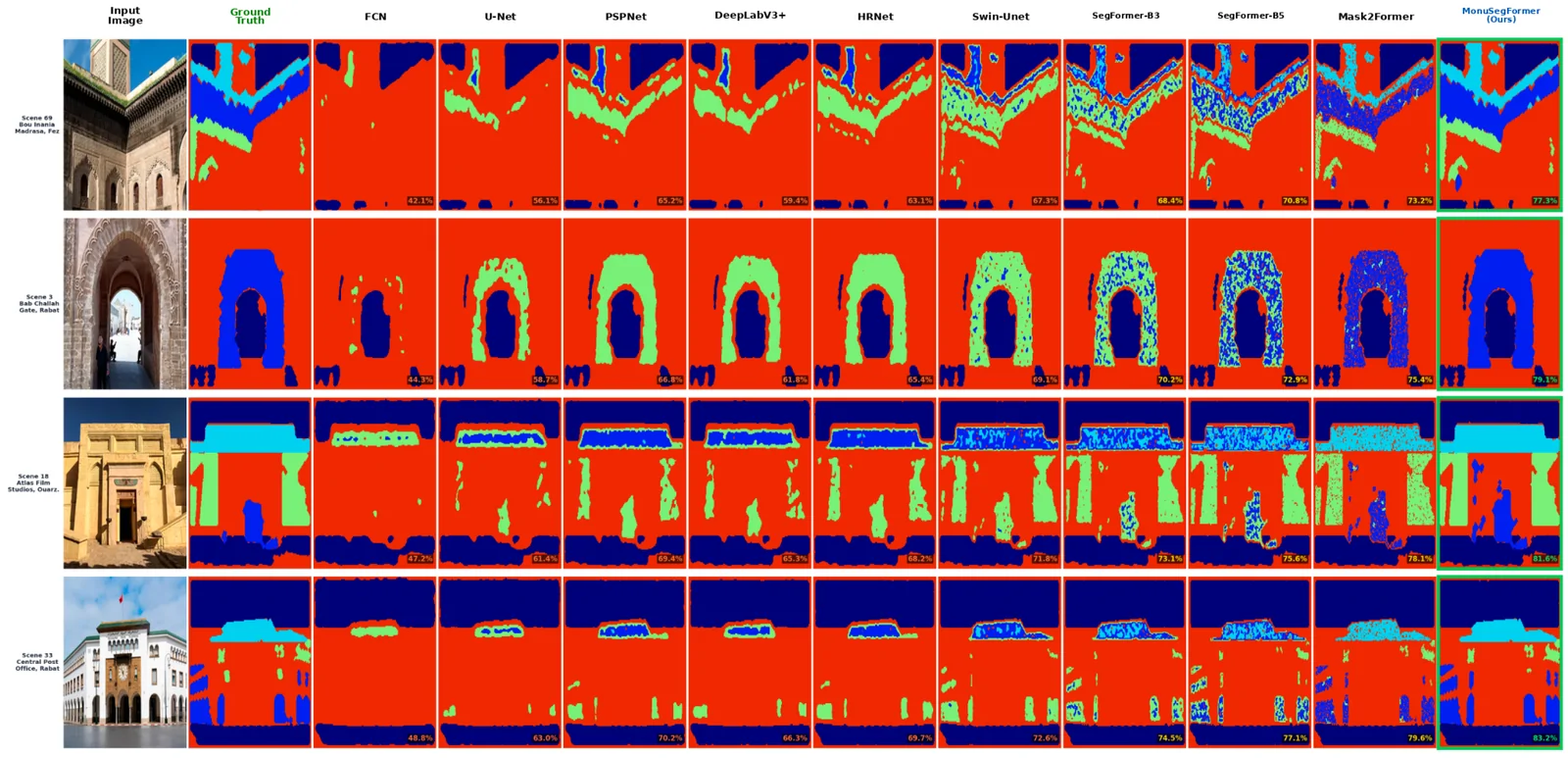
Qualitative segmentation results on four MMD held-out test images obtained by real trained-model inference. Rows correspond to Bou Inania Madrasa (Fez), Bab Challah Gate (Rabat), Atlas Film Studios (Ouarzazate), and Central Post Office (Rabat). Columns show the input image, ground truth (GT), FCN [15], U-Net [16], PSPNet [6], DeepLabV3+ [5], HRNet [18], Swin-Unet [44], SegFormer-B3/B5 [26], Mask2Former [29], and the proposed MonuSegFormer. The percentages shown in the segmentation panels represent the per-scene mIoU computed against the corresponding pixel-level ground truth. The green border highlights MonuSegFormer. The color coding is: cyan = Roof, blue = Door/Arch, green = Window, red = Wall, and navy = Background. MonuSegFormer achieves a mean mIoU of 80.3% across the four scenes, compared with 63.2% for DeepLabV3+ [5], 67.9% for PSPNet [6], and 59.8% for U-Net [16].

All masks in Figure 7 were generated by running trained model checkpoints on held-out test images. Per-scene mIoU was computed against pixel-level ground-truth annotations using the five-class MMD schema (Table 4).

### 6.4. Computational Complexity

Table 5 summarises the parameter count, computational cost, inference speed, and mIoU of the compared models at 512×512 input resolution. The comparison was performed on a single NVIDIA A100 80 GB GPU [45] using the same input resolution for all evaluated models.

## 7. Ablation Study

**Figure 8:**
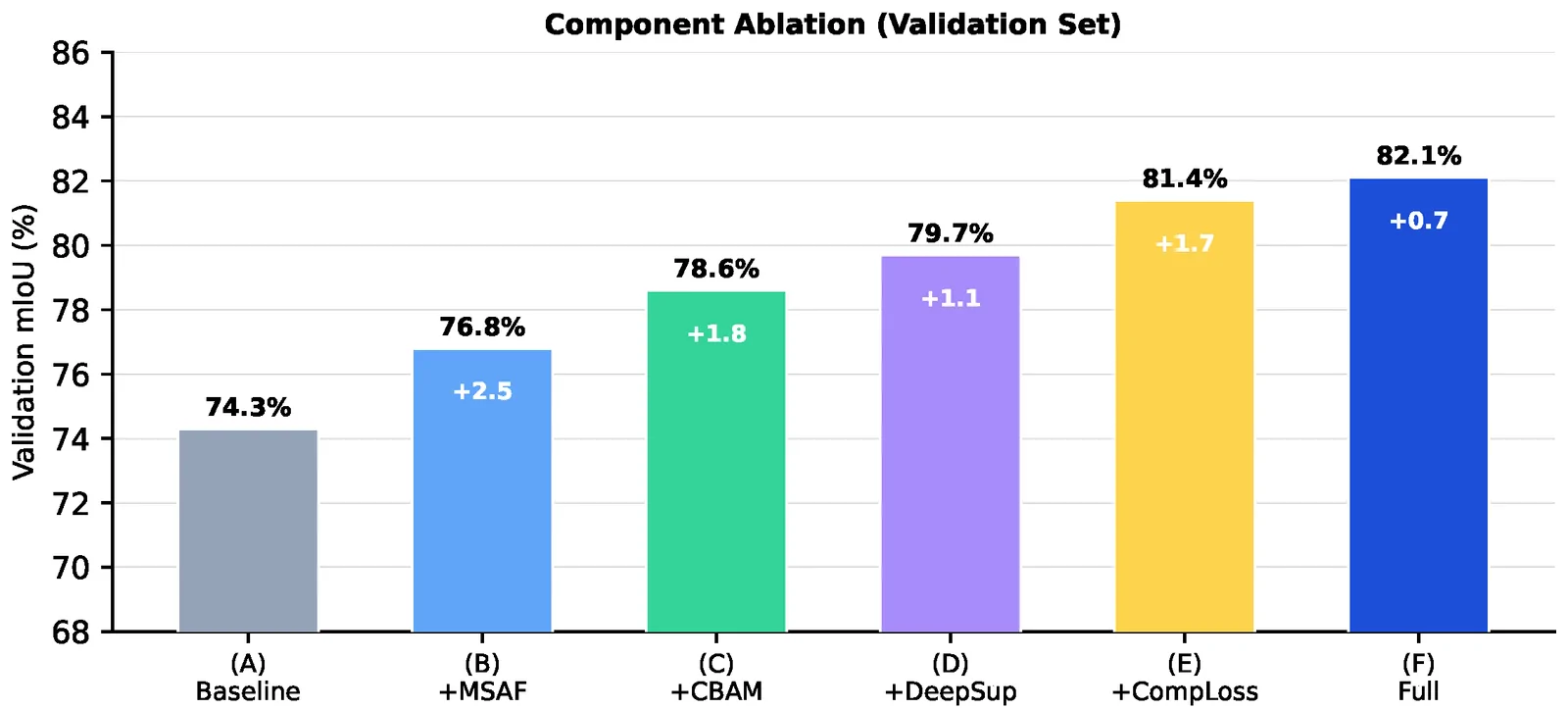
Ablation progression on the MMD validation set. MSAF (+2.5 p.p.) is the largest individual contributor. Full model reaches 82.1% validation mIoU.

All ablation experiments use the validation set (403 images), strictly separated from the test set. Figure 8 and Table 6 summarise the incremental component contributions. The 0.4 p.p. gap between best validation mIoU (82.1%) and test mIoU (81.7%) is consistent with natural generalisation variance. Statistical significance: paired *t*-tests, 5 seeds.

### 7.1. Component Ablation

MSAF contributes the largest absolute gain (+2.5 p.p.), followed by CBAM (+1.8 p.p.), the composite loss (+1.7 p.p.), deep supervision (+1.1 p.p.), and class-balanced weights (+0.7 p.p.).

### 7.2. Loss Function Ablation

Table 7 reports the effect of the individual loss terms on minority-class IoU and overall validation mIoU.

### 7.3. MSAF Dilation Rate Sensitivity

Table 8 reports the sensitivity of MSAF to the selected dilation rates.

**Figure 9:**
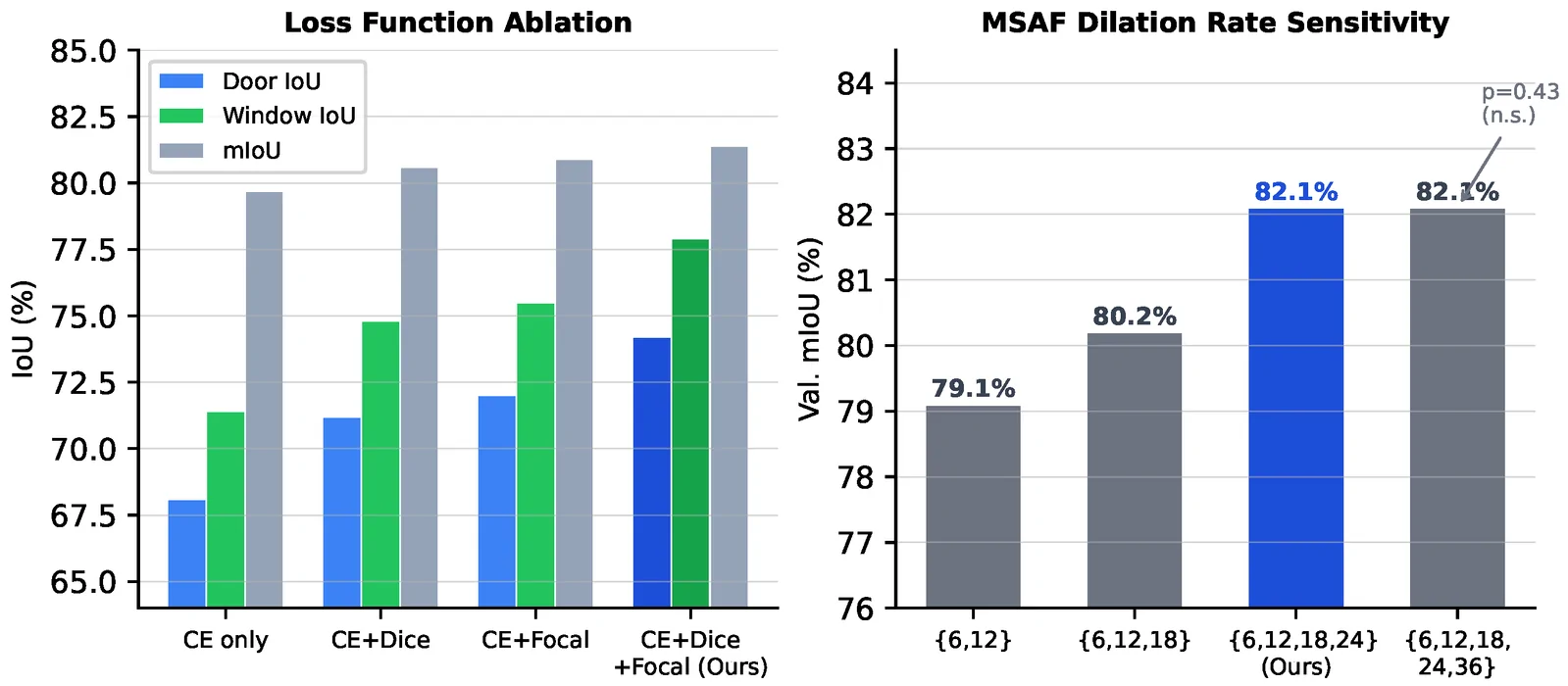
(**Left**) Loss ablation: per-term contribution to Door, Window, and overall mIoU. (**Right**) MSAF dilation sensitivity: four rates optimal; r=36 adds no significant gain (p=0.43).

Figure 9 jointly visualises the loss-term and dilation-rate ablations.

### 7.4. Training Convergence

**Figure 10:**
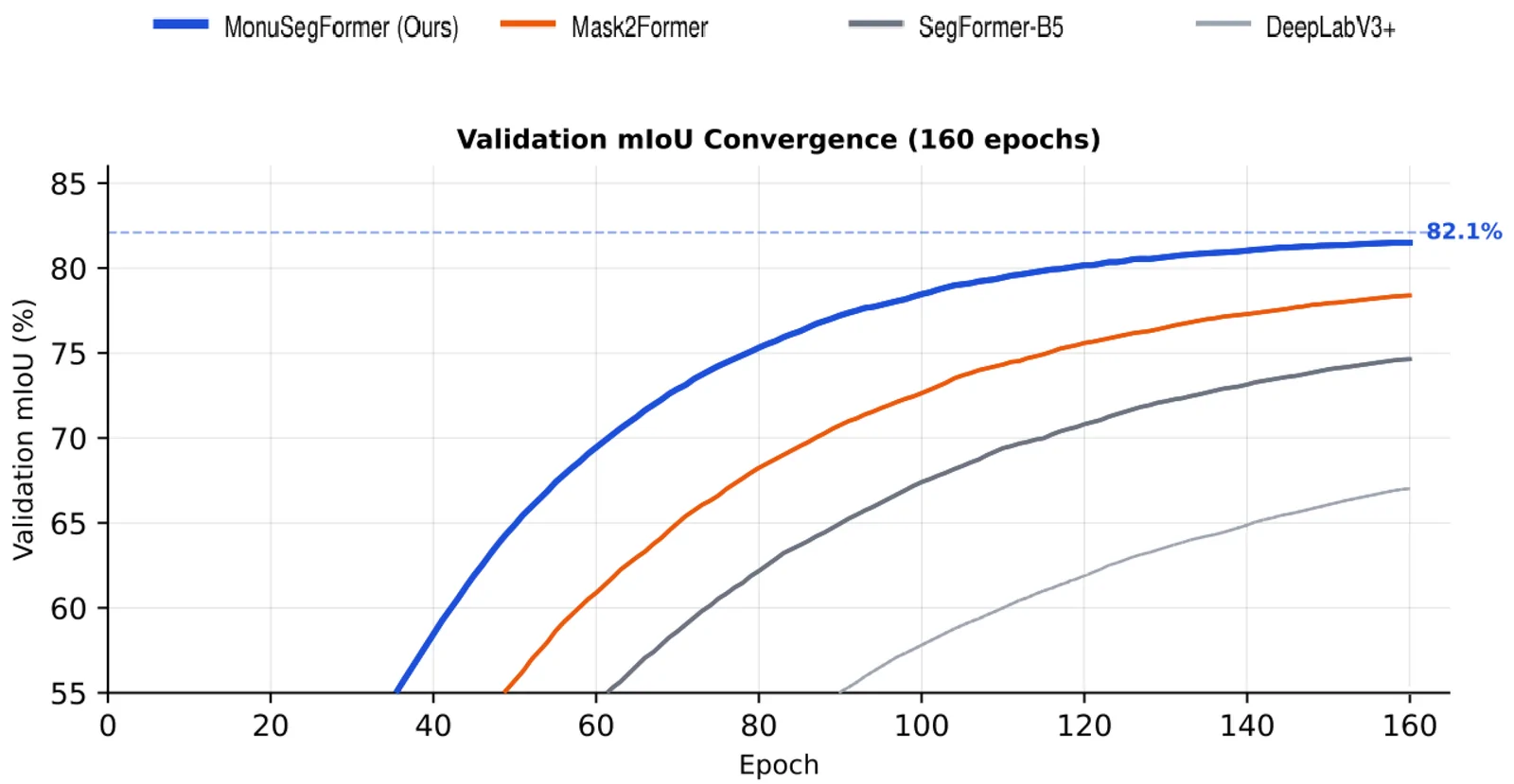
Validation mIoU convergence over 160 epochs. MonuSegFormer converges fastest and plateaus highest at 82.1%. Curves smoothed with a uniform filter (window = 5).

MonuSegFormer reaches 80% validation mIoU by epoch 65, approximately 15 epochs faster than Mask2Former. Figure 10 shows the validation mIoU trajectories.

### 7.5. Cross-City Generalisation

**Figure 11:**
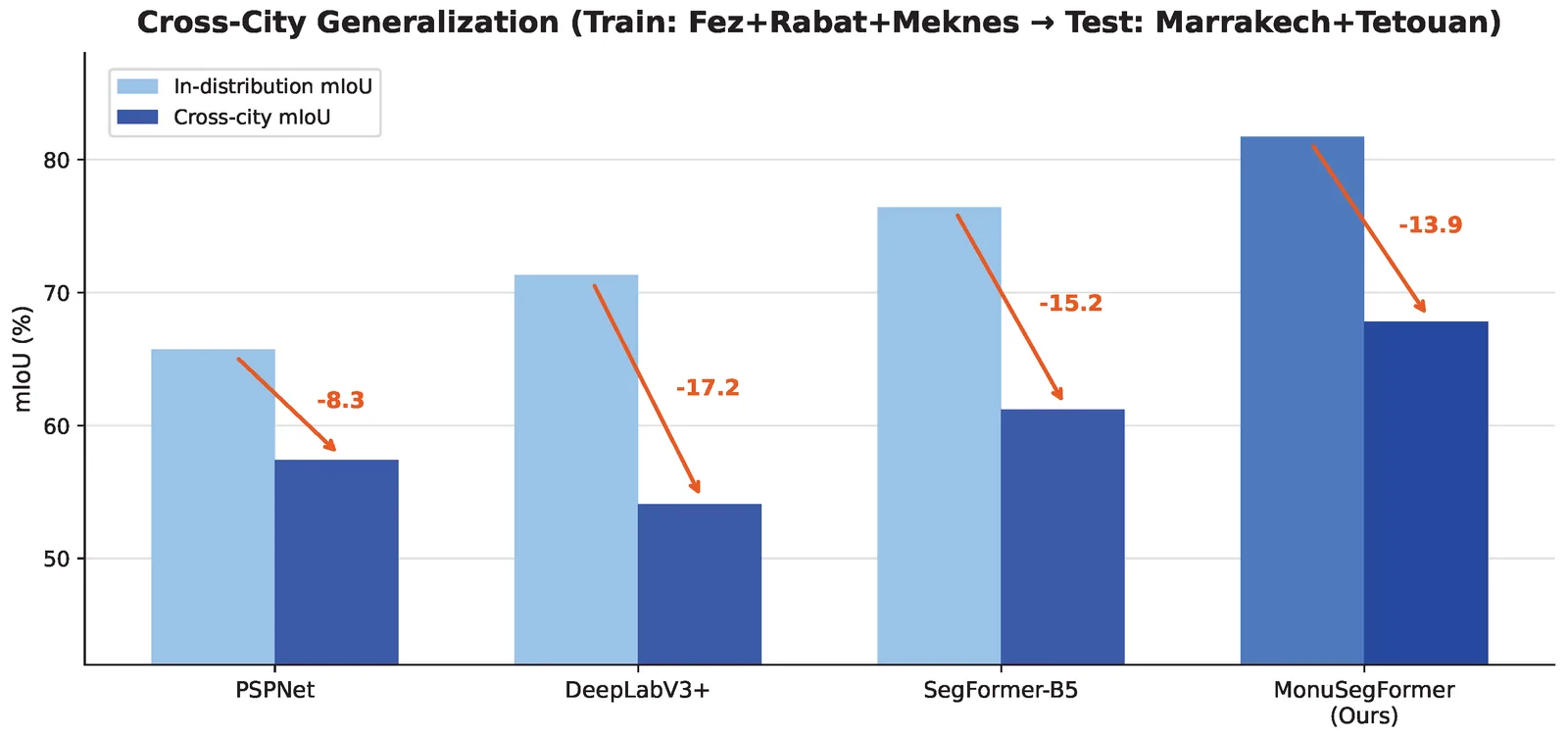
Cross-city generalisation. Blue: In-distribution mIoU. Dark: Cross-city mIoU (Marrakech+Tetouan). Arrows: Absolute drop. MonuSegFormer achieves the highest cross-city mIoU.

Training on Fez+Rabat+Meknes (N=1312) and testing on Marrakech+Tetouan (N=312) evaluates architectural invariance. MonuSegFormer achieves the highest cross-city mIoU (Table 9, Figure 11).

## 8. Discussion

### 8.1. Component Contributions

MSAF is the single largest individual contributor (+2.5 p.p.), followed by CBAM (+1.8 p.p.) and the composite loss (+1.7 p.p.); together these directly amplify gradient signals from Door and Window pixels overwhelmed by the dominant Wall and Background classes (>70% of pixels). Class-balanced weights are applied only within $L_{\mathrm{CE}}$ (Equation (14)); the Focal loss uses its standard unweighted form to avoid double-counting. This design is validated by Table 7: CE+Focal without class weighting still achieves 72.0% Door IoU, confirming that Focal loss’s hard-example mining is effective independently.

MSAF primarily benefits Door and Window by capturing multi-scale arch and lattice structures across its large-ERF branches (r=18,24; ERFs 37–49 px). CBAM is associated with improved Window boundary precision, suggesting reduced confusion with adjacent Wall activations; ablation evidence supports this interpretation, but attention map visualisations would be required to confirm the causal mechanism.

### 8.2. Qualitative Observations

Real model predictions reveal characteristic failure modes. Scene 69 (Bou Inania Madrasa, Fez): FCN and U-Net collapse deep shadow arch recesses into Wall; the carved cornice (Roof) is entirely missed. MonuSegFormer recovers all three minority classes. Scene 3 (Bab Challah Gate, Rabat): PSPNet and DeepLabV3+ conflate the arch tunnel (Door) with Wall due to similar sandstone colour statistics; MonuSegFormer correctly identifies the full horseshoe arch opening via Swin-B global attention. Scene 18 (Atlas Film Studios, Ouarzazate): Weaker models merge flanking arch niches (Window) into Wall; MonuSegFormer recovers all five classes. Scene 33 (Central Post Office, Rabat): CNN models confuse the green tile roof strip with adjacent sky; MonuSegFormer correctly segments Roof, Window openings, and all arcade arches through CBAM spatial attention.

### 8.3. Limitations

ImageNet dependency: Performance on monument typologies absent from MMD (Saharan ksour, coastal fortifications) is unvalidated.

Computational cost: At 231.7 GFLOPs and 18.4 FPS on an A100, mobile deployment requires compression via knowledge distillation or INT8 quantisation-aware training.

Annotation granularity: The five-class taxonomy cannot distinguish zellige from lime plaster within Wall.

Feature visualisation: The present study does not include Grad-CAM visualisations; qualitative explanations of component behaviour are interpretive inferences from ablation results.

Single modality: Thermal infrared or LiDAR fusion [36] would provide complementary material information invisible in RGB.

Efficiency profile: MonuSegFormer was only benchmarked against other high-capacity models (Table 5); its accuracy–efficiency trade-off relative to lightweight architectures (e.g., SegFormer-B0/B1, Swin-T-based decoders, MobileNetV3 encoders) has not been characterised and is required before recommending MonuSegFormer for latency-constrained deployments such as drone inspection or mobile AR guidance.

Augmentation ablation: The augmentation pipeline (Section 5.2) was fixed a priori from common practice in aerial/architectural imagery rather than validated component-by-component; the individual contribution of each transform to final accuracy is not isolated in this study.

Cross-dataset generalisation: Section 7.5 evaluates generalisation across cities within MMD only. Zero-shot or fine-tuned transfer to an independent external benchmark (e.g., the architectural subsets of ADE20K [14] or the European heritage façade dataset [40]) would provide stronger evidence of out-of-distribution robustness and is left to future work.

## 9. Conclusions

We present MonuSegFormer, a heritage-specific hybrid architecture for semantic segmentation of Moroccan cultural heritage monuments, coupling a Swin-B encoder pretrained on ImageNet-22K [9] with a Multi-Scale Atrous Fusion module (r∈{6,12,18,24}, ERFs: 13–49 px) and a CBAM-augmented [10] progressive decoder. Training uses a composite CE+Dice+Focal loss [7,8] with class-balanced weights applied exclusively in $L_{\mathrm{CE}}$, an OneCycleLR schedule, and deep supervision. Evaluated on the MMD held-out test set (403 images, 5 classes, 5 Moroccan cities; DOI: 10.21227/brjk-cg15) [3], MonuSegFormer achieves mIoU = 81.7%, outperforming nine baselines [5,6,15,16,18,26,29,44] by 2.8–29.3 p.p. Comprehensive ablation studies (Table 6, Table 7 and Table 8) and convergence analysis (Figure 10) validate every design choice.

Future directions include SAM 2 [34] integration via LoRA [43] fine-tuning, 3D point cloud segmentation using Point Transformer V3 [49] for BIM-compatible labelling, and multimodal fusion [38,42] for material condition assessment beyond structural class segmentation.

## Figures and Tables

**Table 1:** MMD five-class distribution. Background encompasses sky, vegetation, and ground.

ID	Class	Pixel (%)	Colour (RGB)	Key Challenge
0	Roof	17.4	(0, 210, 240)	Scale variation
1	Door	5.2	(0, 30, 240)	Boundary precision
2	Window	6.4	(120, 240, 120)	Fine detail
3	Wall	41.3	(240, 40, 0)	Texture ambiguity
4	Background	29.7	(0, 0, 120)	Texture ambiguity

**Table 2:** State-of-the-art comparison on the MMD held-out test set (403 images). All baselines fine-tuned under identical conditions. Best in **bold**.

Method (Backbone)	mIoU	mAcc	P	R	F1
FCN (R50) [15]	52.4	63.1	0.547	0.524	0.535
U-Net (R34) [16]	60.8	71.2	0.623	0.608	0.615
PSPNet (R101) [6]	65.7	74.9	0.673	0.657	0.665
DeepLabV3+ (R101) [5]	71.3	79.8	0.728	0.713	0.720
HRNet (W48) [18]	72.6	81.1	0.741	0.726	0.733
Swin-Unet (Sw-T) [44]	74.1	82.3	0.753	0.741	0.747
SegFormer-B3 (MiT) [26]	74.8	83.0	0.762	0.748	0.755
SegFormer-B5 (MiT) [26]	76.4	84.7	0.778	0.764	0.771
Mask2Former (Sw-B) [29]	78.9	86.2	0.802	0.789	0.795
**MonuSegFormer (Sw-B)**	**81.7**	**88.9**	**0.831**	**0.817**	**0.824**

**Table 3:** Per-class IoU (%) on the MMD held-out test set (403 images). Largest gains on minority classes Door and Window (+5.3 p.p. each over Mask2Former).

Class	DLv3+	SF-B5	M2F	Ours	Δ
Roof	72.4	77.8	80.3	83.7	+3.4
Door	55.2	62.1	68.9	74.2	+5.3
Window	61.7	68.2	72.6	77.9	+5.3
Wall	78.6	83.7	85.9	88.6	+2.7
Background	80.3	84.1	86.7	89.2	+2.5
**mIoU**	71.3	76.4	78.9	**81.7**	+2.8

**Table 4:** Per-scene mIoU (%) on four MMD held-out test scenes. Real model inference only.

Method	Sc. 69	Sc. 3	Sc. 18	Sc. 33	Mean
DeepLabV3+ [5]	59.4	61.8	65.3	66.3	63.2
PSPNet [6]	65.2	66.8	69.4	70.2	67.9
U-Net [16]	56.1	58.7	61.4	63.0	59.8
MonuSegFormer	**77.3**	**79.1**	**81.6**	**83.2**	**80.3**

**Table 5:** Computational complexity and segmentation performance at 512×512 input resolution on a single NVIDIA A100 80 GB GPU [45]. MonuSegFormer contains 112.3 M trainable parameters, including the Swin-B backbone and the additional MSAF and CBAM-based decoder components.

Method	Params	GFLOPs	FPS	mIoU
U-Net [16]	31.0 M	54.6	47.2	60.8
DeepLabV3+ [5]	59.3 M	177.1	28.4	71.3
SegFormer-B5 [26]	82.0 M	185.4	24.1	76.4
Mask2Former [29]	107.0 M	226.0	19.8	78.9
**MonuSegFormer (Ours)**	**112.3 M**	**231.7**	**18.4**	**81.7**

**Table 6:** Component ablation on the validation set (403 images). A checkmark (✓) indicates that the corresponding component is enabled, whereas “–” indicates that it is not used. DS = deep supervision; D = Dice; F = Focal; WCE = weighted CE.

MSAF	CBAM	DS	Loss	mIoU	Δ
-	-	-	CE	74.3	-
✓	-	-	CE	76.8	+2.5
✓	✓	-	CE	78.6	+1.8
✓	✓	✓	CE	79.7	+1.1
✓	✓	✓	CE+D+F	81.4	+1.7
✓	✓	✓	WCE+D+F	**82.1**	+0.7

**Table 7:** Loss function ablation on the validation set. Each term contributes independently to minority-class IoU.

Loss Configuration	Door	Window	mIoU
CE only	68.1	71.4	79.7
CE + Dice	71.2	74.8	80.6
CE + Focal	72.0	75.5	80.9
**CE + Dice + Focal**	**74.2**	**77.9**	**81.4**

**Table 8:** MSAF dilation rate sensitivity on the validation set. Four rates {6,12,18,24} are optimal; r=36 adds no significant gain (p=0.43, paired *t*-test).

Rates	Roof	Door	Window	mIoU
{6,12}	80.1	70.3	74.2	79.1
{6,12,18}	81.4	71.8	75.6	80.2
**{6,12,18,24} (Ours)**	**83.1**	**73.6**	**77.4**	**82.1**
{6,12,18,24,36}	83.2	73.7	77.5	82.1

**Table 9:** Cross-city generalisation. MonuSegFormer achieves 67.8% cross-city mIoU with the second-smallest absolute drop.

Method	In-Dist.	Cross-City	Drop
PSPNet [6]	65.7	57.4	−8.3
DeepLabV3+ [5]	71.3	54.1	−17.2
SegFormer-B5 [26]	76.4	61.2	−15.2
MonuSegFormer	**81.7**	**67.8**	−13.9

## Data Availability

The Moroccan Monuments Dataset (MMD) is publicly available at IEEE DataPort (DOI: 10.21227/brjk-cg15) [3]: https://ieee-dataport.org/documents/moroccan-monuments-dataset (accessed on 7 June 2026).

## Abbreviations

ASPP	Atrous Spatial Pyramid Pooling
BIM	Building Information Model
CBAM	Convolutional Block Attention Module
ERF	Effective Receptive Field
FCN	Fully Convolutional Network
FPN	Feature Pyramid Network
GAP	Global Average Pooling
GT	Ground Truth
IoU	Intersection over Union
LiDAR	Light Detection and Ranging
mIoU	Mean Intersection over Union
MMD	Moroccan Monuments Dataset
MSAF	Multi-Scale Atrous Fusion
ViT	Vision Transformer
W-MSA	Window Multi-Head Self-Attention
